# Statins on hepatocellular carcinoma risk in hepatitis B or C patients protocol for a systematic review and meta-analysis

**DOI:** 10.1097/MD.0000000000011950

**Published:** 2018-08-24

**Authors:** Zhiguo Li, Ying Li, Xiaoke Li, Ludan Zhang, Nanqi Zhao, Hongbo Du, Bo Zhou, Yong’an Ye

**Affiliations:** a Department of Gastroenterology, Dongzhimen Hospital affiliated to Beijing University of Chinese Medicine (BUCM); b Beijing University of Chinese Medicine; c Institute of Liver Diseases; d Centre for Evidence-Based Chinese Medicine, BUCM, Beijing, China.

**Keywords:** hepatitis, hepatocellular carcinoma, statins, systematic review

## Abstract

**Background:**

Hepatocellular carcinoma (HCC) is a leading cause of cancer-related death worldwide. Chronic hepatitis B (HBV) and C virus (HCV) infection causes liver cancer. This protocol is to provide the methods used to assess the relationship between statins and HCC risk in hepatitis B or C patients.

**Methods:**

We will search comprehensively the PubMed (medline), Embase, Web of Science, Cochrane Library, and China National Knowledge Infrastructure, Wanfang Database from their inception to November 2017. We will include studies that evaluated and clearly defined exposure to statins, reported the HCC incidence in hepatitis B/C patients or HBV/HCV-related cirrhosis patients, provided effective comparison groups, and reported risk estimates, such as hazard ratios, relative risks, or odds ratios with corresponding 95% confidence intervals or sufficient data for their estimation. We will use Stata (version 15.0) to compute the data synthesis carefully when a meta-analysis is allowed.

**Results:**

This study will provide a high-quality synthesis of current evidence of statins on HCC risk in hepatitis B or C patients.

**Conclusion:**

The conclusion of our systematic review will provide evidence to judge whether statin is an effective intervention for hepatitis B or C patients.

## 1 Introduction

Hepatocellular carcinoma (HCC) remains the third leading cause of cancer deaths worldwide.^[1]^ Chronic infections with hepatitis B (HBV) and C viruses (HCV) are key risk factors for liver cancer.^[2,3]^ HBV is a major global public health problem, affecting approximately 240 million individuals globally.^[4]^ HCV affects more than 185 million individuals worldwide.^[5]^ Antiviral therapies are the preferred first-line medications for patients infected with HBV or HCV. HCC incidence is significantly reduced in patients who achieve HCV clearance or suppression of HBV replication with a sustained virologic response to antiviral therapies.^[6,7]^ While the use of antiviral therapies can be effective etiology-specific HCC chemopreventive interventions, a viral cure does not eliminate HCC risk, especially in patients with cirrhosis or fibrosis.^[2,8]^

Statins, 3-hydroxy-3-methylglutaryl CoA reductase inhibitors, are major cholesterol-lowering drugs and have been used to prevent and treat various cardiovascular diseases. Recently, other potential benefits of statins have attracted increasing worldwide attention. For example, studies have shown that statins can decrease the incidence of some cancers, including prostate,^[9]^ colorectal,^[10]^ and liver^[11–13]^ cancers. Additionally, researchers have observed a relationship between statins and the risk of HCC in hepatitis B or C patients. However, no published meta-analysis has investigated the effect of statins on the risk of HCC.

With the above in mind, we conducted a systematic review and meta-analysis of the relevant literature to better understand the relationship between statins and the risk of HCC in hepatitis B or C patients.

## 2 Methods

This protocol follows the guidelines according to the preferred reporting items for systematic reviews and meta-analysis protocol (PRISMA-P)^[14]^ and the Cochrane Handbook for Systematic Reviews of Interventions. This systematic review protocol has been registered on PROSPERO as CRD42017077142. We will describe the changes in our full review if needed.

### 2.1 Types of studies

Either randomized-controlled trials (RCTs) or observational studies (cohort, nested case-control, or case-control studies) will be included.

### 2.2 Types of patients

Adults (aged 18 years or over) including both males and females who were diagnosed with chronic hepatitis B or C (as diagnosed using any recognised diagnostic criteria) will be included, without the limitation of gender or race.

### 2.3 Types of interventions

Interventions are defined as statins (including atorvastatin, fluvastatin, lovastatin, pravastatin, rosuvastatin, simvastatin) at any dose, duration, and route of administration. The control group could be placebo or nonstatins.

### 2.4 The outcome measures

The primary outcome measure was the incidence of HCC.

### 2.5 Search methods for the identification of studies

Electronic searches. We will search comprehensively the 4 English databases including EMBASE, the Cochrane Library, Medline, Web of Science, and 2 Chinese databases including China National Knowledge Infrastructure and Wanfang Database on computer from their inception to November 2017. According to the instruction of Cochrane handbook, we made detailed strategies. The example search strategy in Table 1 will be used for Medline. This search strategy will be modified and used for the other databases. References of all included trials will be hand searched for additional eligible trials.

**Table 1:**
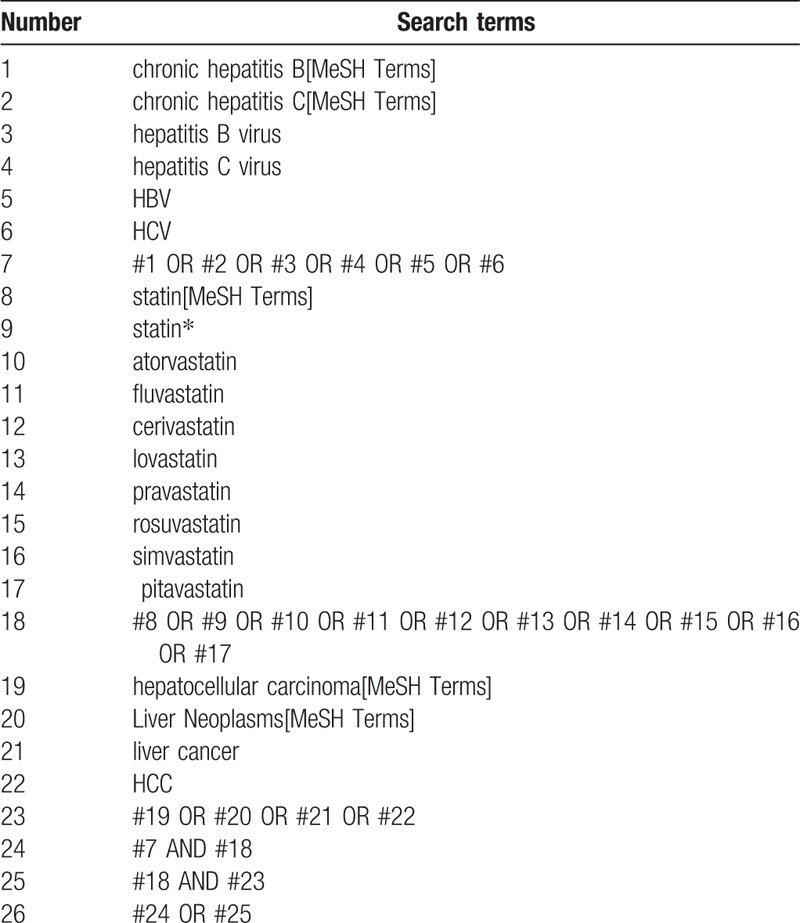
Search strategy used in PubMed database.

### 2.6 Data collection and analysis

#### 2.6.1 Selection of studies

**Figure 1:**
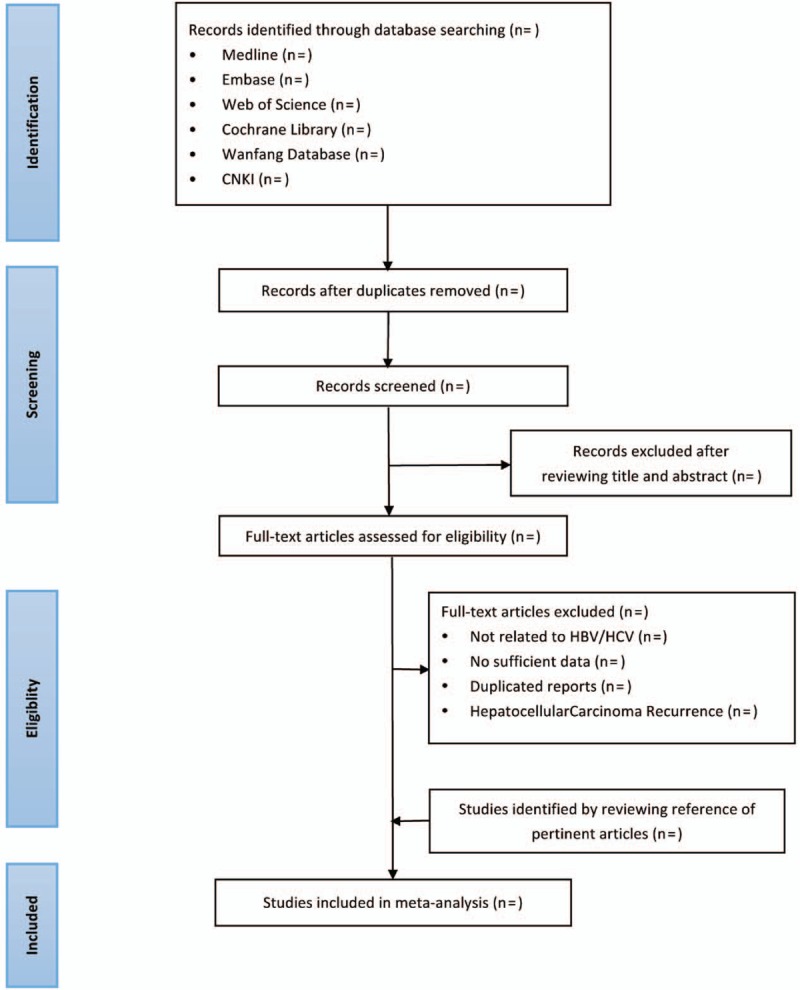
Flow chart of study selection process.

Both searching and the screening will be performed by the 2 reviewers independently. Before selection of studies, all reviewers must get trained to understand the purpose and process of the review. The results will be exported to the Endnote X8 referencing software and duplicate studies will be removed using this software. Initially, we will screen and evaluation the titles and abstracts of studies, and select those likely to be of relevance to our systematic review. In the second stage of selection, full texts will be examined if necessary based on the inclusion criteria. Any disagreements should be resolved through discussion to get a consensus and judged by an arbiter (YY). The study selection procedure is shown in the flow chart (Fig. 1).

#### 2.6.2 Data collection and management

Two independent researchers will extract the basic data and outcome data of included literatures according to the data management table designed in advance: first author, publication time, journal name, study design and key elements of quality evaluation, number of participants (cases, controls, or cohort size), duration of follow-up, comparison of exposure levels, potential adjusted confounding variables, odds ratio /relatitive risk (RR) values and 95% confidence interval (CI) for different categories of statin use. Eventually, another experienced member will deal with the inconsistencies. We will contact the corresponding author for more information if the details of the trials were not completed.

#### 2.6.3 Assessment of risk of bias in included studies

Two independent reviewers will be respondent to assess the risk of bias about eligible studies. If any disagreement is there in assessment, we will reach a consensus via group discussion or consulting with the corresponding author if necessary.

The quality of randomized-controlled studies was assessed according to the Cochrane Handbook for Systematic Reviews of Interventions V.5.1.0. The following 7 domains will be evaluated for the risk of bias, random sequence generation, allocation concealment, blinding of participants and personnel, blinding of outcome assessment, incomplete, outcome data, selective reporting, and other bias. Ultimately, the assessment will be classified into 3 grades: “low risk of bias,” “high risk of biases,” or “unclear risk of bias.”

The quality of observational studies was assessed using the Newcastle–Ottawa Scale.^[15]^ Studies were scored according to 3 items: patient selection (4 stars), comparability of the study groups (2 stars), and assessment of outcome/exposure (3 stars). With this scale (with a maximum of 9 stars), the star rating system was used to indicate the quality of each study. A score of 0 to 6 stars was considered low quality and ≥7 stars high quality.

#### 2.6.4 Measures of treatment effect

The review will use Stata (version 15.0, StataCorp, College Station, TX) to compute meta-analysis when collected data are available. For dichotomous outcomes, the RR will be conducted to indicate extracted data. For measurement data, the mean difference (MD) will be employed correspondingly to data synthesis. A 95% CI will be adopted in either RR or MD to express the effect sizes.

#### 2.6.5 Dealing with missing data

If the required data are not clear or not reported in clinical papers, the reviewers will contact with the original author of the studies via e-mail for complete information. If not, we will analyze available data to perform the outcome; in the meanwhile, we will also assess the potential impact the missing data might cause on the conclusion in the discussion.

#### 2.6.6 Assessment of heterogeneity

Heterogeneity will be assessed by Cochrane's Q statistic (heterogeneity < 0.10 suggesting statistical significance) and the I^2^ statistic. When the I^2^ value is <50%, the study will be considered to have no statistical heterogeneity, and the fixed-effect model will be selected. Although I^2^ ≥ 50%, the study will be considered to have substantial heterogeneity, and we will select a random-effect model. RR will be employed as a common measure of the association between statin use and HCC risk. Both hazard ratios and ORs will be regarded as equivalent to the RR.

#### 2.6.7 Assessment of publication bias

If included trials exceed 10 in the review, we will use funnel plots and statistic test to detect publication bias.

#### 2.6.8 Data synthesis

We will use Stata (version 15.0, StataCorp, College Station, TX) for data synthesis and analysis. When I^2^ < 50%, a fixed-effects model will be used to calculate the RR and MD. When I^2^ ≥ 50%, we will use a random-effects model to synthesize the data. If apparent clinical heterogeneity is demonstrated, the reviewers can carry out the subgroup or sensitivity analysis to explore heterogeneity source including clinical and methodology cause. On the contrary, we only perform descriptive analysis if meta-analysis is not applicable.

#### 2.6.9 Subgroup analysis

Subgroup analysis will be generated if the eligible studies are sufficient (at least 10 trials). With the purpose of exploring the resources of the heterogeneity, we will perform subgroup analysis based on the stage of disease (cirrhosis vs hepatitis) we will take age, sex, race, body mass index, antiviral treatment, cirrhosis, diabetes mellitus (DM), and nonstatin lipid-lowering drugs into account.

#### 2.6.10 Sensitivity analysis

If it is possible, we will proceed a sensitivity analysis to test the robustness of the conclusion, for example, reconduct a meta-analysis and compare with the original one after removing the low quality or small size trial, to explore whether these factors influence the total effect of meta-analysis.

#### 2.6.11 Ethics and dissemination

Ethical approval is not necessary because data used in our study are not linked to individual patient data. Also, the findings will be disseminated through a peer-review publication.

## 3 Discussion

Statins are major cholesterol-lowering drugs, which have been used to prevent and treat various cardiovascular diseases. Studies have shown that statins can decrease the incidence of liver^[11–13]^ cancers. Additionally, researchers have observed a relationship between statins and the risk of HCC in hepatitis B or C patients. However, to our knowledge, whether statin is effective on the risk of HCC in hepatitis B or C patients has not been clearly demonstrated. Therefore, we conduct the review aiming to provide a more leading-edge and objective evidence for clinicians.

We have presented a protocol for a systematic review of statins on HCC risk in hepatitis B or C patients. However, this protocol may have some limitations. First, the use of language including English and Chinese may lead to bias of the study. Second, different dosage of statins, age of patients, and small sample size of trials may induce some bias. We will publish this systematic review in a peer-reviewed journal. If there are the critical changes of this protocol, we will write the changes in the review. This study will form the basis to conduct additional research and provide evidence for statins on HCC risk in hepatitis B or C patients.

## Acknowledgment

The authors thank Dr Jian-ping Liu (Centre for Evidence-Based Chinese Medicine, Beijing University of Chinese Medicine, Beijing, China) for his advice concerning the methodology of the study.

## Author contributions

**Conceptualization:** Zhiguo Li, Yong’an Ye.

**Data curation:** Xiaoke Li, Ludan Zhang, Bo Zhou.

**Funding acquisition:** Yong’an Ye.

**Investigation:** Ludan Zhang, Bo Zhou.

**Methodology:** Zhiguo Li, Ying Li, Xiaoke Li, Nanqi Zhao, Hongbo Du, Yong’an Ye.

**Resources:** Hongbo Du.

**Supervision:** Xiaoke Li, Nanqi Zhao, Hongbo Du, Yong’an Ye.

**Writing – original draft:** Zhiguo Li, Ying Li.
